# Relationship between myomectomy and risk of osteoporosis or fracture: A population-based cohort study

**DOI:** 10.1371/journal.pone.0294405

**Published:** 2023-11-16

**Authors:** Jin-Sung Yuk, Sang-Hee Yoon

**Affiliations:** Department of Obstetrics and Gynecology, Sanggye Paik Hospital, School of Medicine, Inje University, Seoul, Republic of Korea; Harar Health Science College, ETHIOPIA

## Abstract

Myomectomy, a surgery to remove multiple leiomyomas from the uterus, is a treatment option for uterine fibroids (UF) in premenopausal patients. Osteoporosis and bone fractures are known to be strongly associated with menopausal status or hormonal changes. However, no studies have discussed the association between myomectomy and osteoporosis or fractures. This study investigated the risk of osteoporosis or fractures (vertebrae, hip, and others) in Korean patients who had undergone myomectomy without bilateral oophorectomy. We used data from the 10-year claims database of the Korean National Health Insurance from January 2009 to December 2020. Data for patients who had undergone myomectomy without oophorectomy (n = 211,969) and the control group (n = 450,124) who were randomly selected from the database were extracted. The incidence and hazard ratios (HRs) of osteoporosis or fracture between the myomectomy patients and the control group were calculated. A Cox proportional hazards model was used to analyze hazard ratios and 95% confidence intervals (CI). Subgroup analyses were performed based on age. The adjusted hazard ratios for osteoporosis and total fractures were 0.934 (95% CI: 0.916–0.954, P<0.001) and 0.919 (95% CI: 0.896–0.941, P<0.001), respectively, in the myomectomy group. The adjusted hazard ratios according to fracture site were 0.857 (95% CI: 0.799–0.92, P<0.001) for vertebral fractures, 0.706 (95% CI: 0.48–1.037, P = 0.076) for hip fractures, and 0.919 (95% CI: 0.896–0.943, P<0.001) for other fractures. In conclusion, patients who have undergone myomectomy might have a decreased risk of osteoporosis or fractures.

## Introduction

Uterine fibroids (UF) are one of the most common benign tumors in females [1]. These tumors, commonly known as leiomyomas, affect females primarily during their reproductive years, are diagnosed via ultrasonography, and have an estimated prevalence of 4.5–68.6% [2]. Treatment options for UF include medical suppressive therapies (oral contraceptive pills, progesterone receptor modulators, and leuprolide acetate), reductive procedures (uterine artery embolization and radiofrequency ablation), and surgical removal via myomectomy or hysterectomy [3]. Of these, hysterectomy is the most commonly implemented major gynecologic procedure performed in premenopausal patients for UF accompanied by symptoms such as heavy menstrual bleeding, dysmenorrhea, and chronic pelvic pain [4]. Myomectomy, a surgery to remove multiple leiomyomas from the uterus, is another treatment option for premenopausal patients who prefer the option of future childbearing or simply want to conserve their uterus [5].

UF is thought to be associated with female hormones. Females of reproductive age have been reported to have up to a 70% chance of developing UF, which declines rapidly after menopause [6]. A retrospective study reported that patients with UF had higher vertebral bone mineral density (BMD) than that of the reference group [7], while another prospective study presented that perimenopausal and early postmenopausal patients with a history of UF had better BMD and fewer bone fractures compared to those without UF [8]. Since the growth of UF has been known to be dependent on ovarian steroid hormones [9], we can speculate that there is an association between UF and bone health.

Osteoporosis and bone fractures are strongly associated with menopausal status or hormonal changes. The key risk factor for bone loss in middle-aged females is the deficiency of sex-steroid hormones, such as estrogen or testosterone, which decreases with reduced ovarian reserve [10]. Osteoporosis is a disease of the skeletal system in which bone mass is reduced, which disrupts bone structure, causing decreased bone strength and eventually leading to fragility fractures [11]. In addition to increased psychogenic problems and social costs due to functional impairments in daily life activities, fractures are associated with high mortality and morbidity [12].

Some studies have investigated the association between UF and osteoporosis as the occurrence of both diseases might be associated with female hormones. Several studies have explored the association between hysterectomy and osteoporosis or fractures. Previous studies have shown that hysterectomy is associated with an increased risk of osteoporosis and bone fractures [11, 13–15]. It is also thought to be associated with a variety of comorbidities since it might be related to earlier physiological menopause and hormonal changes than the general population, linking them to osteoporosis and bone fractures [16]. Hysterectomy in the premenopausal period harms ovarian function due to decreased ovarian blood flow and follicular atresia [17, 18].

However, no studies have discussed the association between myomectomy and osteoporosis or fractures. This study aimed to investigate the risk of osteoporosis and fractures in patients who underwent myomectomy in South Korea using a national sample cohort from the Korean National Health Insurance Service (NHIS).

## Materials and methods

### Study design and database

By law, all Koreans are required to enroll for the national health insurance [19]. The Korean National Health Insurance Corporation gathers medical health insurance records on the majority of Koreans (approximately 51 million people) and records information such as age, gender, illness, operation name, prescription drug name, type of medical insurance, inpatient and outpatient therapy. To claim medical insurance expenditures, all medical facilities in Korea provide basic information to the Health Insurance Review and Assessment Service (HIRA). The HIRA is an independent national institution that evaluates this data and informs the National Health Insurance Corporation whether or not to pay for the medical expenditures of the patient. As a result, HIRA is the primary repository for the medical records of Korean citizens. Between January 1, 2009, and December 31, 2020, this population-based retrospective cohort study analyzed HIRA insurance data.

### Population identification

The Korea Health Insurance Medical Care Expenses (2016, 2019 version) and The International Classification of Diseases, 10^th^ revision (ICD-10) were adopted in the analysis of this study.

Women between the ages of 20 and 49 with a uterine fibroid diagnosis code (D25.x) and myomectomy (R4121, R4122, R4123, R4124, R4125, R4126, R4127, R4128, R4129) were included in this study. Women between the ages of 20 and 49 who visited a medical facility for health check-ups were chosen as the control group. Cases having the diagnosis code (D25.x) of uterine fibroids were excluded from the control group at least once in the total cohort. Patients who underwent myomectomy or had health exams in 2009 and 2010 were excluded from the washout. Women who had previously attended a medical facility for cancer (Cxx), osteoporosis (M80-M85), or fractures (S02, S12, S22, S32, S42, S52, S62, S72, S82, S92, T02, T08, T10, T12) were excluded from the study.

### Outcomes

When a bone mineral density test (quantitative computed tomography, dual-energy absorptiometry, and radiographic absorptiometry) was performed in conjunction with an osteoporosis diagnosis code or when osteoporosis medications (estrogen, tibolone, bisphosphonate, denosumab, romosozumab, teriparatide, and SERM) were administered in conjunction with the diagnosis code, osteoporosis was defined. A fracture was defined as two or more visits to a medical facility as a result of other fractures (S02/S01.8-S01.9/S22.2-S22.9/S32.3-S32.8/S42/S52/S62/S82/S92/T02/T08/T10/T12), vertebral fractures (S12.7/S22.0-S22.1/S32.0-S32.2), hip fractures (S72).

### Confounding variables

Age, socioeconomic status (SES), and region of residence, at the time, of the study participants were the confounding variables used in this study. Age was classified at 5-year intervals, and the subject was classified as having a low SES when medical insurance was medical aid. Rural regions were described as residents of non-metropolitan areas. Charlson comorbidity index (CCI) was estimated using diagnostic codes covering the period from inclusion to one year ago [20]. Parity was determined by analyzing whether or not delivery happened within the cohort. Hypertension (I10-15), diabetes mellitus (E10-14), hyperlipidemia (E78), and menopause (N95, N80.0, M81.0, E28.3) were defined as having the corresponding disease when the patient visited a medical institution more than twice with each corresponding diagnosis code. If adnexal surgery extirpation of adnexal tumor, ovarian wedge resection, incision and drainage of ovarian cyst, or adhesional adnexectomy was conducted more than once, it was considered to have been performed. If menopausal hormone medication (estrogen, tibolone), steroid, or calcium/vitamin D supplements were used for more than six months before inclusion, they were classified as menopausal hormone therapy (MHT) before inclusion, steroid before inclusion, or calcium/vitamin D supplements before inclusion, respectively. If it was utilized following inclusion, it was determined as a variable following inclusion.

### Statistics

R 3.0.2 (The R Foundation for Statistical Computing) and SAS Enterprise Guide 6.1 (SAS Institute Inc.) were used to conduct all statistical analyses. For all statistics, a two-sided test was used, and a p-value of 0.05 or less was considered statistically significant. Pearson’s Chi-square or Fisher’s exact test was used to analyze categorical variables, while a t-test or Mann–Whitney U-test was used to analyze continuous variables. After adjusting for confounding variables, Cox regression analysis was used to evaluate the risk of osteoporosis or fracture. The inclusion day was chosen as the starting point for Cox analysis, and the end date was chosen as the date of death or December 31, 2020. The listwise deletion approach was used when the missing value was less than 10%, while the regression imputation method was used when the missing value was greater than 10%. Cox regression analysis was performed on women residing in urban regions to confirm the robustness of our study results.

### Ethics

The study was approved by the Institutional Review Board of Sanggye Paik Hospital (Approval number: SGPAIK 2021-07-003). HIRA altered the variable values used to identify people to anonymize the participants. Therefore, informed consent was not required under the Bioethics and Safety Act of South Korea. Due to the privacy policy set by HIRA, the raw data saved on the server is not accessible to anybody other than researchers. In addition, the HIRA servers were required to conduct study analysis and results. Although this study makes use of HIRA data, HIRA has no interest in the study.

## Results

### Subject characteristics

**Fig 1:**
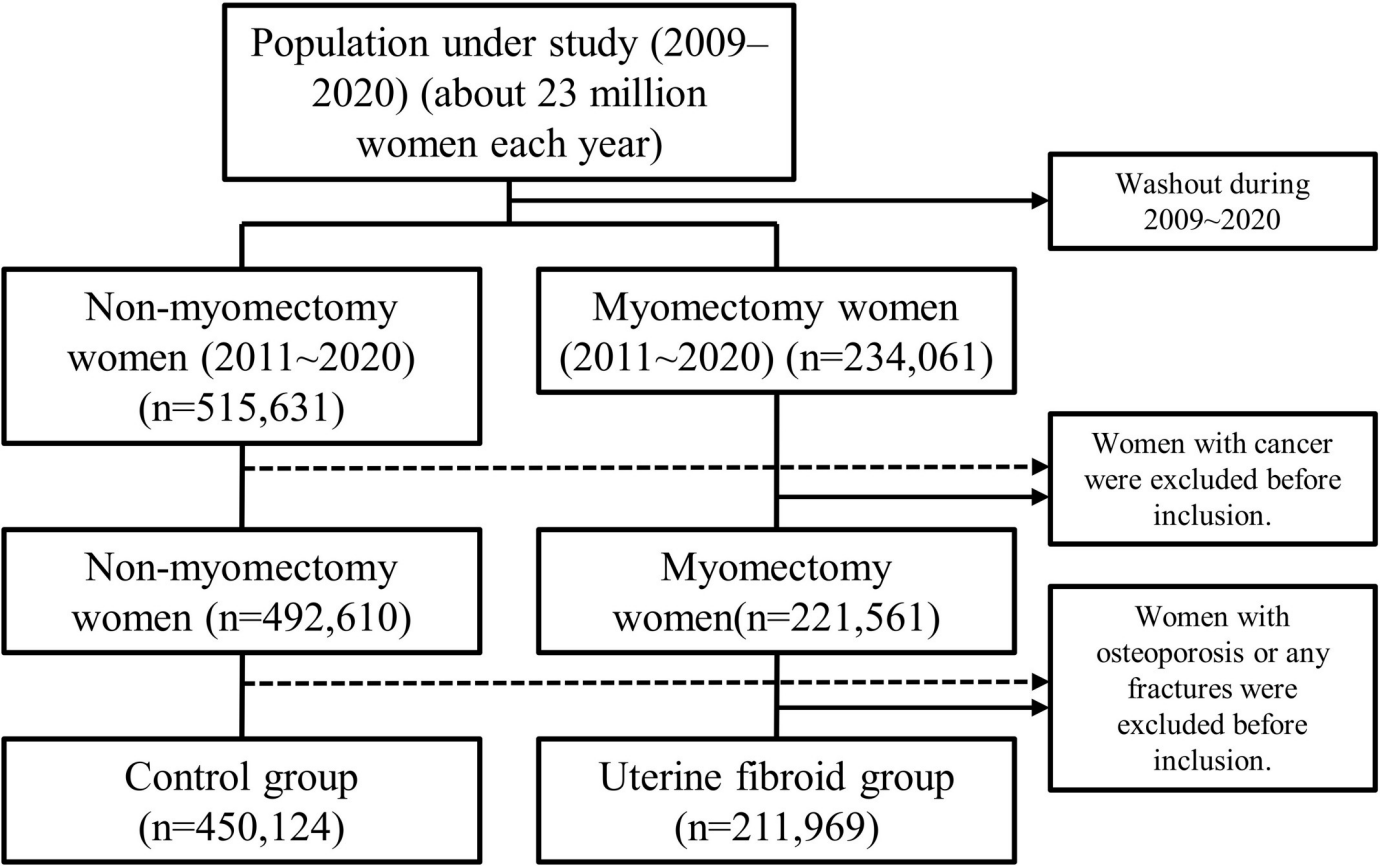
Study flowchart to identify women based on myomectomy from the National Health Insurance Database (2011–2020).

Fig 1 shows the flowchart representing the methodology used to recruit the myomectomy and control groups from the National Health Insurance Research Database (NHIRD). The study, myomectomy, and control cohorts included 662,093, 211,969, and 450,124 women, respectively (Fig 1). The median ages of the UF and control cohorts were 40 and 35 years, respectively (Table 1). The UF cohort had a higher prevalence of hypertension and dyslipidemia at baseline compared with the control cohort (P < 0.001).

**Table 1:** Basic characteristics of the participants according to whether or not a myomectomy was performed obtained from the National Health Insurance Database (2011–2020).

	Control	Uterine fibroid	Total	P-value
Number of women	450,124	211,969	662,093	
Median age (years)	35 [28–42]	40 [35–45]	37 [30–43]	<0.001
Median follow-up period (years)	4 [1.6–6.7]	4.8 [2.5–7.3]	4.3 [1.9–6.9]	<0.001
Age at inclusion (years)				<0.001
20∼24	55,396 (12.3)	1,960 (0.9)	57,356 (8.7)	
25∼29	74,613 (16.6)	12,712 (6)	87,325 (13.2)	
30∼34	91,262 (20.3)	33,132 (15.6)	124,394 (18.8)	
35∼39	76,941 (17.1)	47,298 (22.3)	124,239 (18.8)	
40∼44	74,104 (16.5)	62,951 (29.7)	137,055 (20.7)	
45∼49	77,808 (17.3)	53,916 (25.4)	131,724 (19.9)	
SES				<0.001
Mid-high SES	440,689 (97.9)	209,717 (98.9)	650,406 (98.2)	
Low SES	9,435 (2.1)	2,252 (1.1)	11,687 (1.8)	
Region				<0.001
Urban area	237,091 (52.7)	132,489 (62.5)	369,580 (55.8)	
Rural area	213,033 (47.3)	79,480 (37.5)	292,513 (44.2)	
CCI				<0.001
0	356,470 (79.2)	168,173 (79.3)	524,643 (79.2)	
1	62,188 (13.8)	26,873 (12.7)	89,061 (13.5)	
≥2	31,466 (7)	16,923 (8)	48,389 (7.3)	
Parity in cohort				<0.001
0	343,750 (76.4)	190,528 (89.9)	534,278 (80.7)	
1	70,666 (15.7)	13,884 (6.6)	84,550 (12.8)	
≥2	35,708 (7.9)	7,557 (3.6)	43,265 (6.5)	
Hypertension before inclusion				<0.001
Absent	426,970 (94.9)	197,961 (93.4)	624,931 (94.4)	
Present	23,154 (5.1)	14,008 (6.6)	37,162 (5.6)	
DM before inclusion				0.085
Absent	430,494 (95.6)	202,921 (95.7)	633,415 (95.7)	
Present	19,630 (4.4)	9,048 (4.3)	28,678 (4.3)	
Dyslipidemia before inclusion				<0.001
Absent	391,070 (86.9)	181,116 (85.4)	572,186 (86.4)	
Present	59,054 (13.1)	30,853 (14.6)	89,907 (13.6)	
Menopause before inclusion				<0.001
Absent	435,859 (96.8)	207,510 (97.9)	643,369 (97.2)	
Present	14,265 (3.2)	4,459 (2.1)	18,724 (2.8)	
MHT before inclusion				<0.001
Absent	445,472 (99)	210,416 (99.3)	655,888 (99.1)	
Present	4,652 (1)	1,553 (0.7)	6,205 (0.9)	
Steroid before inclusion				<0.001
Absent	397,512 (88.3)	190,264 (89.8)	587,776 (88.8)	
Present	52,612 (11.7)	21,705 (10.2)	74,317 (11.2)	
Calcium Vitamin D supplements before inclusion				<0.001
Absent	447,950 (99.5)	211,113 (99.6)	659,063 (99.5)	
Present	2,174 (0.5)	856 (0.4)	3,030 (0.5)	
Adnexal surgery before inclusion				<0.001
Absent	438,680 (97.5)	208,275 (98.3)	646,955 (97.7)	
Present	11,444 (2.5)	3,694 (1.7)	15,138 (2.3)	

CCI, Charlson comorbidity index; DM, diabetes mellitus; MHT, menopausal hormone therapy; SES, socioeconomic status.

Data are expressed as the number (%) or median [25 percentile, 75 percentile].

### Risk of osteoporosis or fracture

Table 2 shows the incidence of osteoporosis and fractures in patients who underwent myomectomy as compared to the control cohorts. Overall, the incidence of osteoporosis and bone fractures in patients who underwent myomectomy was significantly lower than that in the control group.

**Table 2:** Osteoporosis and fracture incidences post-myomectomy.

	Control	Uterine fibroid	Total	P-value
Number of women	450,124	211,969	662,093	
Osteoporosis				<0.001
Absent	424,041 (94.2)	195,366 (92.2)	619,407 (93.6)	
Present	26,083 (5.8)	16,603 (7.8)	42,686 (6.4)	
Vertebral fracture				0.013
Absent	447,579 (99.4)	210,665 (99.4)	658,244 (99.4)	
Present	2,545 (0.6)	1,304 (0.6)	3,849 (0.6)	
Hip fracture				0.591
Absent	450,030 (100)	211,929 (100)	661,959 (100)	
Present	94 (0)	40 (0)	134 (0)	
Other fracture				<0.001
Absent	431,448 (95.9)	202,004 (95.3)	633,452 (95.7)	
Present	18,676 (4.1)	9,965 (4.7)	28,641 (4.3)	
Total fracture				<0.001
Absent	429,287 (95.4)	200,841 (94.8)	630,128 (95.2)	
Present	20,837 (4.6)	11,128 (5.2)	31,965 (4.8)	

Data are expressed as the number (%).

**Fig 2:**
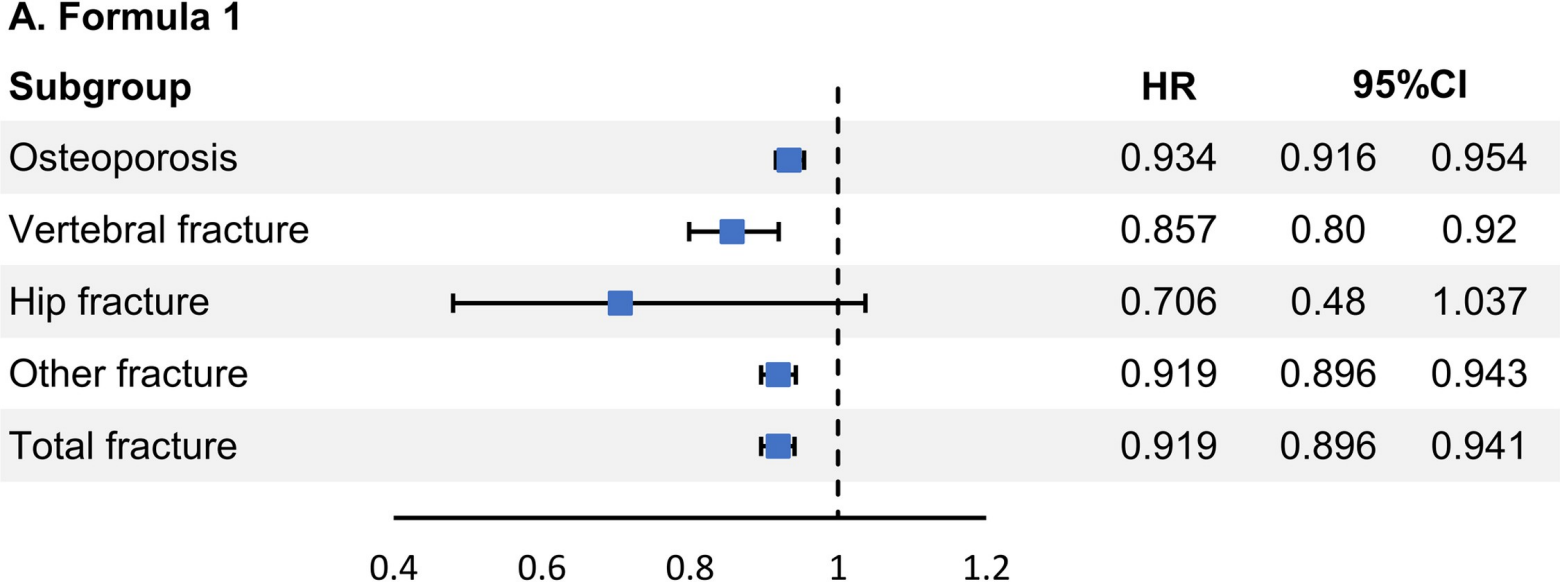
Subgroup analysis to present hazard ratios according to the fracture site.

Table 3 shows the HR of osteoporosis and fractures between the UF and control cohorts, stratified according to several variables, namely, age, socioeconomic status (SES), region, Charlson comorbidity index (CCI), parity, hypertension, diabetes mellitus (DM), dyslipidemia, menopause before inclusion, menopausal hormone therapy (MHT) before inclusion, steroid before inclusion, calcium—vitamin D supplements before inclusion, and adnexal surgery before inclusion. Overall, the risk of osteoporosis and fractures in patients who underwent myomectomy was significantly lower than that in the control group [adjusted HR (aHR) = 0.934, 95% confidence interval (CI) = 0.916–0.954 and adjusted HR (aHR) = 0.919, 95% CI = 0.896–0.941, respectively]. Fig 2 presents the HR according to the fracture site. For vertebral fractures, myomectomy was associated with a lower risk than the control cohort (aHR = 0.857, 95% CI = 0.799–0.92). For hip fractures, myomectomy had no lower risk than the control cohort (aHR = 0.706, 95% CI = 0.48–1.037). For other bone fractures, the risk in patients who underwent myomectomy was lower than that in the control cohort (aHR = 0.919, 95% CI = 0.896–0.943).

**Table 3:** Hazard ratios for osteoporosis and fractures in women with and without myomectomy.

	Unadjusted		Adjusted ^a^	
	HR (95% CI) ^a^	P-value	HR (95% CI) ^a^	P-value
Osteoporosis				
Reference (no uterine myoma)	1 (reference)		1 (reference)	
Uterine fibroid	1.148 (1.125–1.17)	<0.001	0.934 (0.916–0.954)	<0.001
Vertebral fracture				
Reference (no uterine myoma)	1 (reference)		1 (reference)	
Uterine fibroid	0.915 (0.856–0.987)	0.009	0.857 (0.799–0.92)	<0.001
Hip fracture				
Reference (no uterine myoma)	1 (reference)		1 (reference)	
Uterine fibroid	0.748 (0.517–1.083)	0.124	0.706 (0.48–1.037)	0.076
Other fracture				
Reference (no uterine myoma)	1 (reference)		1 (reference)	
Uterine fibroid	0.955 (0.932–0.979)	<0.001	0.919 (0.896–0.943)	<0.001
Total fracture				
Reference (no uterine myoma)	1 (reference)		1 (reference)	
Uterine fibroid	0.956 (0.934–0.978)	<0.001	0.919 (0.896–0.941)	<0.001

CCI, Charlson comorbidity index; CI, confidence interval; DM, diabetes mellitus; HR, hazard ratio; MHT, menopausal hormone therapy; SES, socioeconomic status.

^a^ HRs were adjusted for myomectomy, age, SES, region, CCI, parity, hypertension, DM, dyslipidemia, menopause before inclusion, MHT before inclusion, steroid before inclusion, calcium vitamin D supplements before inclusion, adnexal surgery before inclusion.

### Subgroup analyses of the risk of osteoporosis or fractures with age

Subgroup analyses were performed based on age (Table 4). Participant age was defined as the age at the time of myomectomy. Compared with the control cohort, the age-specific risk of osteoporosis in the UF cohort was significantly higher in patients aged 30–39 years, with an aHR of 1.311 (95% CI = 1.218–1.412). However, the age-specific risk of osteoporosis in the UF cohort was significantly lower in patients aged 40–49 years with an aHR of 0.867 (95% CI = 0.849–0.885).

**Table 4:** Hazard ratios for osteoporosis and fractures in women with and without myomectomy by age group.

	20–29 years ^a^		30–39 years ^a^		40–49 years ^a^	
	HR (95% CI) ^a^	P-value	HR (95% CI) ^a^	P-value	HR (95% CI) ^a^	P-value
Osteoporosis						
Reference (no uterine myoma)	1 (reference)		1 (reference)		1 (reference)	
Uterine fibroid	1.027 (0.737–1.433)	0.873	1.311 (1.218–1.412)	<0.001	0.867 (0.849–0.885)	<0.001
Vertebral fracture						
Reference (no uterine myoma)	1 (reference)		1 (reference)		1 (reference)	
Uterine fibroid	1.003 (0.758–1.328)	0.981	0.788 (0.688–0.902)	<0.001	0.846 (0.776–0.922)	<0.001
Hip fracture						
Reference (no uterine myoma)	1 (reference)		1 (reference)		1 (reference)	
Uterine fibroid	0.63 (0.08–4.947)	0.661	1.345 (0.58–3.121)	0.49	0.592 (0.377–0.93)	0.023
Other fracture						
Reference (no uterine myoma)	1 (reference)		1 (reference)		1 (reference)	
Uterine fibroid	0.904 (0.822–0.994)	0.038	0.928 (0.887–0.972)	<0.001	0.902 (0.873–0.932)	<0.001
Total fracture						
Reference (no uterine myoma)	1 (reference)		1 (reference)		1 (reference)	
Uterine fibroid	0.912 (0.833–0.999)	0.047	0.909 (0.87–0.949)	<0.001	0.908 (0.88–0.937)	<0.001

CCI, Charlson comorbidity index; CI, confidence interval; DM, diabetes mellitus; HR, hazard ratio; MHT, menopausal hormone therapy; SES, socioeconomic status.

^a^ HRs were adjusted for myomectomy, age, SES, region, CCI, parity, hypertension, DM, dyslipidemia, menopause before inclusion, MHT before inclusion, steroid before inclusion, calcium vitamin D supplements before inclusion, adnexal surgery before inclusion.

Patients with myomectomy had a lower risk of fracture than those in the control cohort in the age groups 20–29 years (aHR = 0.912, 95% CI = 0.833–0.999), 30–39 years (aHR = 0.909, 95% CI = 0.87–0.949), and 40–49 years (aHR = 0.908, 95% CI = 0.88–0.937). For vertebrae fractures, the patients who underwent myomectomy had a lower risk than the control cohort in the 30–39 years (aHR = 0.788, 95% CI = 0.688–0.902) and 40–49 years (aHR = 0.846, 95% CI = 0.776–0.922) age groups. For hip fractures, patients who underwent myomectomy only had a lower risk than the control cohort in the 40–49 age group (aHR = 0.592, 95% CI = 0.377–0.93). Among all age groups, the risk of other bone fractures was lower in patients who underwent myomectomy than in the control cohort.

## Discussion

Based on the data obtained from the HIRA-NIS, we found that patients who underwent myomectomy had a lower incidence of osteoporosis (HR, 0.934; 95% CI = 0.916–0.954; P <0.001). In the subgroup analysis with age, the HR of osteoporosis was significantly greater in patients who had undergone myomectomy in the 30–39 years age group, unlike in the other subgroup. This study reported that patients who underwent myomectomy at any age had a lower incidence of total fractures (HR, 0.919; 95% CI = 0.896–0.941; P <0.001). In our study, we found a nearly 14.3% decreased risk of vertebral fractures and an 8.1% decreased risk of other fractures in the myomectomy group. However, there was no significant decrease in hip fractures.

The low incidence of hip fractures in middle-aged patients might explain these results. According to a worldwide study, the incidence of hip fracture peaks between the ages of 75 and 79 years, although all fractures are most common between the ages of 50 and 59 years, with the incidences decreasing with age [21]. A similar pattern in the change in risk of fracture was shown in another study [11]. Regarding the fracture site, vertebral fracture (aHR = 4.67, 95% CI = 3.59–6.08, P <0.001) and other bone fractures (aHR = 3.91, 95% CI = 3.44–4.45, P <0.001) were higher in the hysterectomized patients than in the control group. In contrast, hip fracture (aHR = 0.92, 95% CI = 0.55–1.54, P = 0.74) was not significantly higher.

Regarding osteoporosis, modifiable risk factors include smoking, excess alcohol consumption, inadequate intake of calcium and vitamin D, and low body weight, while non-modifiable risk factors include age, race, and early menopause [22].

The development of UF involves complex and heterogeneous factors. The concentrations of steroid hormones (especially, estrogens and progesterone) and growth factors (insulin-like growth factor-I, transforming growth factor-β, fibroblast growth factor, and epidermal growth factor) play a pivotal role in the development and growth of UF [23]. Estrogen and progesterone influence UF development by regulating growth factors and their signaling pathways [24]. Based on a recent review, intrinsic abnormalities of the myometrium, abnormal myometrial receptors for estrogen, hormonal changes, and altered responses to ischemic damage during the menstrual period may play a role in the initiation of the epigenetic and genetic changes found in these tumors [23]. Furthermore, recent meta-analysis studies on the relationship between the risk of UF and smoking or drinking have shown that cigarette smoking and alcohol consumption are not associated with UF risk [25, 26]. However, a subgroup analysis including only three cohort studies found a slightly reduced risk of UF in current and former smokers [25]. In two subgroup analyses comparing current alcohol drinkers to abstainers, current alcohol drinkers had a slight increase in the risk of UF [26]. Given the limited number of studies and potential implications, further research on smoking and drinking is needed.

As both the uterus and bone are hormone-responsive organs, it is conceivable that the mechanisms linking UF and osteoporosis may be related to female sex hormones. In general, UF is thought to be hormonally responsive to estrogen. For instance, they tend to grow upon exposure to estrogens or during pregnancy [8], and may regress after menopause or during gonadotropin-releasing hormone agonist treatment, leading to a hypo-estrogen environment [27].

Bone mass is typically influenced by endogenous and exogenous estrogens [28]. For instance, hyperestrogenism, which may occur in obese patients or postmenopausal hormone replacement therapy, tends to be inversely associated with the risk of osteoporosis or fractures [29, 30]. Therefore, the hyperestrogenic states existing with UF may induce a protective effect against bone loss and fractures.

In patients with severe symptoms due to uterine fibroids or in those who had failed medical treatment, surgical treatment was considered. Transient ischemia during a hysterectomy can affect growing follicles, leading to a temporary decrease in the anti-Mullerian hormone (AMH) level until new follicles are recruited [31]. AMH is produced by the granulosa cells of growing follicles within the ovaries and is typically used to quantify the ovarian reserve [32]. The surgical procedure of incising the uterus and removing leiomyoma tissue might interrupt blood flow to the ovaries, resulting in a temporary decrease in AMH level [31]. A prospective study that assessed 35 patients who were undergoing hysterectomy found that serum AMH was significantly lower at 2 days and 3 months post-hysterectomy as compared to pre-operative level [33]. In a national sample cohort study, the incidence of osteoporosis was found to have increased in patients who had undergone hysterectomy as compared to matched controls, regardless of bilateral oophorectomy status (HR, 1.45; 95% CI, 1.37–1.53; P, <0.001) [16]. Another retrospective population-based cohort study reported that patients who underwent hysterectomy might be associated with increased risks of developing osteoporosis (HR, 1.44; 95% CI, 1.28–1.61; P, <0.001) or vertebral fractures (HR, 4.67; 95% CI, 3.59–6.08; P, <0.001) [11]. An early decrease in sex hormone levels resulting from reduced AMH in hysterectomized patients may cause earlier bone loss and increase the risk of osteoporosis as compared to patients with an intact uterus. Two prospective studies have shown that myomectomy is associated with a transient decline in AMH levels in the immediate postoperative period; however, it was similar to the preoperative level 3 months after surgery [31, 33]. If the ovarian function does not decrease after myomectomy, the high estrogenic status in patients with existing uterine fibroids may positively affect the reduction of osteoporosis and fractures.

However, in addition to a decrease in ovarian function, there are other factors to consider when performing myomectomy. The introduction of laparoscopic morcellation allows for minimally invasive treatment of uterine pathology [34] but raises the risks of recurrence and death due to tumor cells spreading from unknown sarcomas [35]. Uterine leiomyosarcoma is a rare and highly malignant neoplasm that develops from the endometrial connective tissue or the myometrium [35]. Generally, sarcomas are aggressive and have a prognosis worse than other uterine tumors [36]. However, as it is not easy to determine whether a uterine tumor is benign or malignant prior to the operation, diagnosis is typically made after the hysterectomy or myomectomy for the presumed benign leiomyomas. Distinguishing between these diagnoses requires pathologic analysis of the resected specimen, characterized by the presence of severe nuclear atypia, necrosis of tumor type, and high mitotic index [23, 37]. Hence, the misdiagnosis of uterine leiomyosarcoma is a major concern, particularly after the use of laparoscopic morcellation. In fact, the US Food and Drug Administration issued a warning in 2020 to prohibit the use of laparoscopic power morcellators in gynecologic surgery for suspected malignancy [38].

To the best of our knowledge, this is the first population-based cohort analysis focusing on the relationships between myomectomy and the risk of osteoporosis or fractures. Our findings clarify that myomectomy has negative effects on bone health. Among the several risk factors mentioned simultaneously for UF and osteoporosis, we believe that female hormones, in particular, play a significant role in the development of UF and the reduction of osteoporosis. By addressing concerns regarding bone health, our study may alleviate concerns about the long-term side effects of surgical treatment for UF in patients undergoing myomectomy. However, when performing myomectomy, we should not only strive to preserve ovarian function but also minimize the possibility of misdiagnosing uterine sarcoma, which has been reported to have a worse prognosis when tissue is removed through morcellation. This study may be helpful for further research on the impact of different myomectomy methods, such as vaginal, laparotomic, and laparoscopic myomectomy, on bone health. Our study can be further substantiated by accounting for potential confounders and including additional data in the future.

There are some potential limitations to this study based on a review of electronic medical records. First, although the control group was matched for medical history and demographic factors, some possible confounders such as obesity, smoking, alcohol intake, family history of osteoporosis, and other medical diseases that affect bone mass were not considered. Laboratory data including calcium, magnesium, and phosphate levels were not collected from the database. Second, the actual fracture incidence rate may not be accurately reflected since fractures were assigned according to the number of treatment visits using health insurance claims data. Third, it is challenging to distinguish between osteoporotic fractures and fractures caused by other etiologic factors by using diagnosis codes for fractures.

Nonetheless, our study has several strengths. To our knowledge, based on a nationwide population-based cohort analysis, our study is the first to evaluate the risk of osteoporosis and bone fractures in patients who undergo myomectomy. Since our study is a population-based assessment, its study design can minimize selection bias. Additionally, the control group was constituted from an inventory of the Korean population. The study data were adjusted for traditional medical histories, including CCI, parity, hypertension, diabetes mellitus, dyslipidemia, menopause status, menopausal hormone therapy, steroid intake, and calcium/vitamin D supplement intake. The CCI score is one of the widely used clinical indices for various disorders and cancers [39]. Furthermore, a subgroup analysis was performed on age and fracture sites considered to be the two major leading sites of osteoporotic fractures, the hip and vertebra.

## Conclusions

Myomectomy may be associated with a slightly decreased risk of osteoporosis and bone fractures in middle-aged patients. Our findings suggest that myomectomy does not negatively affect bone health. In the future, further studies using an intricate matching process are required.

## Supporting information

S1 Checklist STROBE (Strengthening The Reporting of OBservational studies in Epidemiology) checklist. (PDF)
